# Golden ratio and self-similarity in swimming: breast-stroke and the back-stroke

**DOI:** 10.3389/fnhum.2023.1176866

**Published:** 2023-07-24

**Authors:** Cristiano M. Verrelli, Cristian Romagnoli, Nicolò Colistra, Ivo Ferretti, Giuseppe Annino, Vincenzo Bonaiuto, Vincenzo Manzi

**Affiliations:** ^1^Department of Electronic Engineering, University of Rome Tor Vergata, Rome, Italy; ^2^Sport Engineering Lab, Department of Industrial Engineering, University of Rome Tor Vergata, Rome, Italy; ^3^Department of Human Science and Promotion of Quality of Life, San Raffaele Open University, Rome, Italy; ^4^Biomechanical and Video-Analysis Area for the National Teams of “Federazione Italiana Nuoto”, Rome, Italy; ^5^Human Performance Lab, Centre of Space Biomedicine, Department of Medicine Systems, University of Rome Tor Vergata, Rome, Italy; ^6^Department of Humanities Science, Pegaso Open University, Naples, Italy

**Keywords:** swimming, self-similarity, golden ratio, Fibonacci sequence, neuroscience

## Abstract

**Introduction:**

Dynamics-on-graph concepts and generalized finite-length Fibonacci sequences have been used to characterize, from a temporal point of view, both human walking & running at a comfortable speed and front-crawl & butterfly swimming strokes at a middle/long distance pace. Such sequences, in which the golden ratio plays a crucial role to describe self-similar patterns, have been found to be subtly experimentally exhibited by healthy (but not pathological) walking subjects and elite swimmers, in terms of durations of gait/stroke-subphases with a clear physical meaning. Corresponding quantitative indices have been able to unveil the resulting hidden time-harmonic and self-similar structures.

**Results:**

In this study, we meaningfully extend such latest findings to the remaining two swimming strokes, namely, the breast-stroke and the back-stroke: breast-stroke, just like butterfly swimming, is highly technical and involves the complex coordination of the arm and leg actions, while back-stroke is definitely similar to front-crawl swimming. An experimental validation with reference to international-level swimmers is included.

## 1. Introduction

Very recent research directions have been devoted to providing a theoretical foundation to the experimental evidence that human movements, such as walking and running, are able to induce time-harmonic motor patterns. The resulting findings have shown that such harmonic structures are characterized by the golden ratio occurring as the ratio of the durations of the walking and running gait sub-phases that composed of generalized Fibonacci sequences (Marino et al., 2020; Verrelli et al., 2021a). Most surprisingly, even front-crawl and butterfly swimming behave, from this point of view, like walking and running at middle/long-distance pace (Verrelli et al., 2021b,c). Even in these cases, harmonically self-similar temporal partitions can be formally defined, with quantitative indices being accordingly defined. On the other hand, experimental data on front-crawl and butterfly swimmers have definitely shown how the self-similarity level increases with the swimming technique, while an enhanced self-similarity (namely, a stronger version of the self-similarity) is especially linked to the performance of top-level swimmers. Now, studies on neurophysiology have found that locomotor movements come from the interrelationship between cortical input, central pattern generators, and sensory feedback. With this respect, the middle/long-distance pace in swimming is the mirrored counterpart of the comfortable speed in walking. At such a speed, locomotor systems save energy, whereas their activity is only required to oppose gravity and keep posture, as well as to reintegrate energy losses. On the other hand, the swimming technique is crucially involved (see the related discussion in Verrelli et al. (2021b) and references therein) in learning the complex movements that counteract buoyancy, weight, thrust, and drag. In this light, high/top-level athletes are the ones who are actually able to avoid redundant time- and energy-consuming movements while achieving in-phase synchronization with induced water waves (see the related discussion in Verrelli et al. (2021b) and references therein).

Since the above analysis is, nowadays, restricted to front-crawl and butterfly swimming, the aim of this study is to extend such latest findings to the remaining two swimming strokes, namely, back-stroke and breast-stroke. While back-stroke seems to be similar to front-crawl swimming [both of them exhibit alternating upper and lower limb motions and body roll around the longitudinal axis (Seifert and Chollet, 2009; Psycharakis and Sanders, 2010; Gonjo et al., 2018)], breast-stroke, just like butterfly swimming, is highly technical due to the complex coordination of the arm and leg actions.

Indeed, breast-stroke is one of the most challenging swimming strokes because of the discontinuity between the propulsive actions of arms and legs and its complex time synchronization (Soares et al., 1999). Breast-stroke efficiency is associated with an increased level of propulsive continuity and thus with more highly coordinated motor actions (Sanders, 1996). In flat breast-stroke coordination, the propulsive phase of one limb typically happens when the glide phase of the other occurs. On the other hand, the arm and leg recovery phases almost simultaneously occur (Persyn et al., 1979). Now, comparative assertions regarding swimming performance and coordination can be certainly formulated once quantitative indices have been suitably defined. Chollet et al. (2000) have been the first authors to provide the first pertinent index of coordination for the arms' motion in front-crawl, which is defined as the ratio of the lag time between the start of propulsion by one arm and the end of propulsion by the other one. Such a study has encouraged the scientific community to formulate a new practical indicator for describing the complex arm-leg coordination in breast-stroke similar to the one concerning the front-crawl stroke. The importance of studying and analyzing the arm-leg coordination in breast-stroke has induced by Takagi et al. (2004) to investigate the technique of the world's current top-level breast-strokers during racing. The purpose of their study has been to analyze differences in stroke phases, arm-leg coordination, and intra-cycle hip velocity fluctuation in breast-stroke due to race events for both competitive male and female swimmers. The results have pointed out that the highest-level breast-strokers typically adopt a longer non-propulsive glide phase. Thus, the non-propulsive phase plays a key role in achieving the best performance. Takagi et al. have also demonstrated the importance of a long glide phase in top-level breast-strokers by analyzing the intra-cycle hip velocity fluctuations. They have underlined how the best swimmers must avoid decelerating rapidly during the non-propulsive glide phase by resorting to a low-resistance posture and stroke. Indeed, high variations of intra-cycle velocity generally impose high energy costs while reducing performance (Vilas-Boas, 1996). Therefore, breast-strokers should consider how to minimize the fluctuations of intra-cycle hip velocity during the non-propulsive glide phase. While Colman et al. (1998) have compared the intra-cycle velocity fluctuations of flat and undulation breast-stroke styles, in another study, Seifert and Chollet (2007) have proposed new practical indices of arm/leg coordination and propulsion to be successfully used by coaches and swimmers. They have analyzed differences between men and women in flat breast-stroke arm and leg coordination over different race paces and have introduced a new index of flat breast-stroke propulsion, which represents a measurement of the total duration of arm and leg propulsion. Moreover, they have introduced four time intervals (T1, T2, T3, and T4), which represent the temporal gaps between the arm and leg stroke phases. Such intervals are expressions of inter-limb coordination and assess the level of continuity between the propulsive phases of the arm and leg. Seifert and Chollet (2007) have discovered that elite swimmers present shorter temporal gaps and longer body propulsion with increasing velocity. Thus, the highest technique and skills in breast-stroke are related to the most highly synchronized arm and leg recoveries and increased continuity between propulsion phases. A higher degree of continuity in the propulsion actions implies more highly coordinated motor patterns and thus, better performance. Several studies have shown how male and female breast-stroke swimmers exhibit a higher stroke rate and a lower stroke length in the 100-m event than in the 200-m event (Pai et al., 1984; Chollet et al., 1996; Thompson et al., 2000). Unlike other swimming strokes, stroke rate has been shown to be the most discriminative feature of velocity in the breast-stroke (Pai et al., 1984; Chollet et al., 1996). Some studies (Clarys, 1979; Kolmogorov and Duplischeva, 1992) have suggested that the ratio of stroke rate to stroke length, the velocity, and the active drag are more dependent on swimming technique and thus, on the inter-limb coordination, than on anthropometric characteristics. In their study, Seifert and Chollet (2007) have discovered how men typically show shorter temporal gaps, body glide, and recovery but higher body propulsion with respect to women. Men have shown propulsive actions with greater continuity and overlap between arm glide and leg insweep. Such a strategy of superposition of actions allows high-level swimmers, especially in the sprint race, to reduce speed fluctuations and body deceleration while maintaining a high mean speed within the whole race. Moreover, it has been shown how the superposition of arm and leg coordination allows swimmers to overcome active drag and swim faster. This finding is in line with the study mentioned in the reference, in which, Vilas-Boas (1996) has suggested that the flat breast-stroke is more economical than the undulated style being characterized by the over-water recovery of the arms.

This study, then, extends the last contributions regarding the self-similar pattern-based quantitative indices for front-crawl/butterfly strokes to the breast-stroke, with a forecast on the backstroke. Experimental results involving international-level swimmers are included.

## 2. Methods

### 2.1. Breast-stroke: phase partition

**Figure 1:**
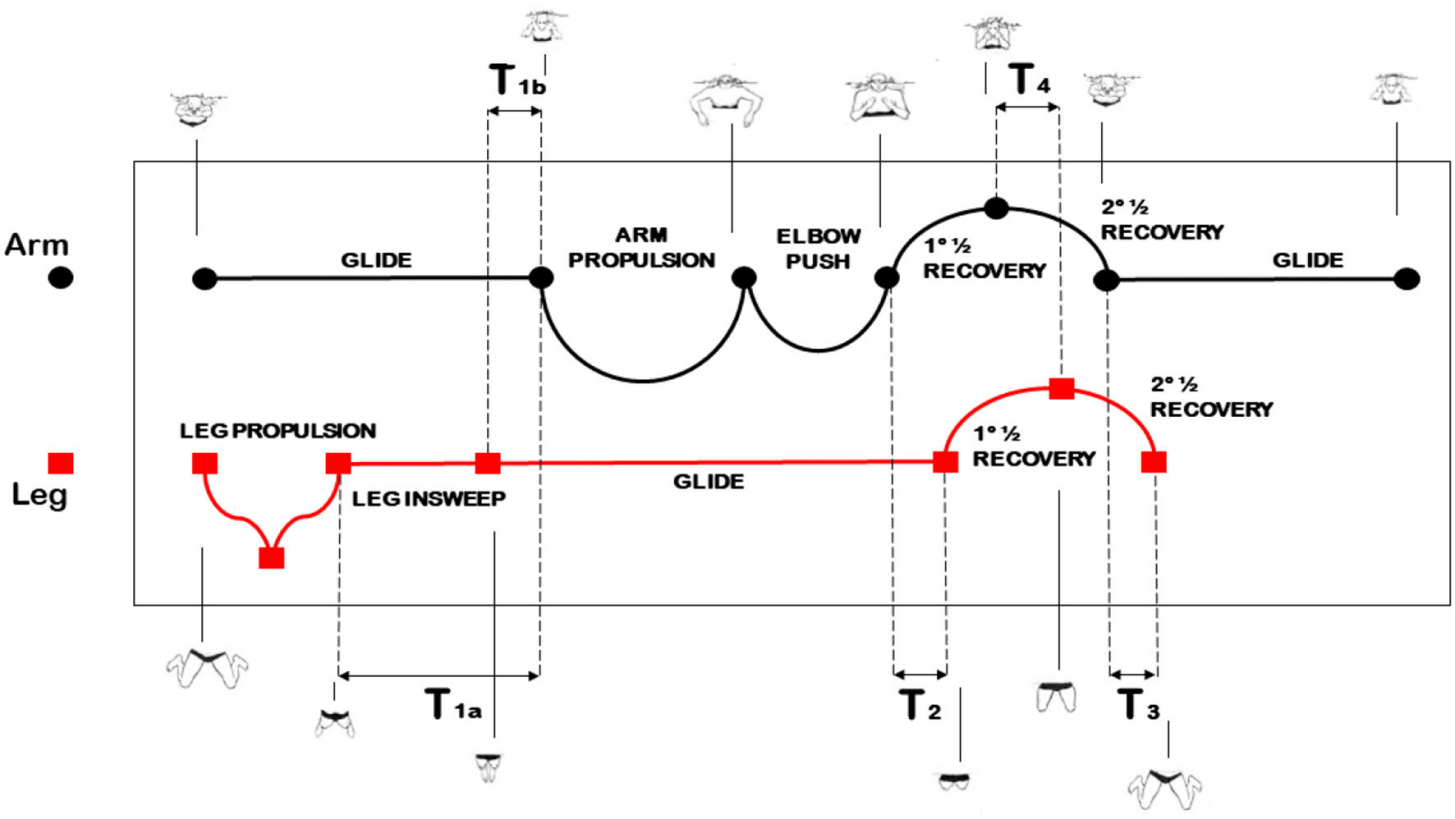
Illustrative arm and leg phase-partition in the breast-stroke.

According to the breast-stroke phase-partition mentioned in Seifert and Chollet (2007), the arm stroke is divided into five phases (Figure 1):

- *arm glide*, with duration AG: phase between the arm extension and the beginning of the hand backsweep.
- *arm propulsion*, with duration AP: phase between the beginning and the end of the hand backsweep.
- *elbow push*, with duration EP: phase between the end of the hand backsweep and the beginning of the forward hand drive.
- *first part of the recovery*, with duration REC1: phase between the end of the elbow push and the arm recovery up to an arm/forearm angle of 90 deg.
- *second part of the recovery*, with duration REC2: phase between the end of the first part of the recovery and the extension.

The sum of the above durations provides the duration S of the arm stroke.^1^ The leg stroke consists of five phases (Figure 1, again):

- *leg propulsion*, with duration LP: phase between the beginning of the backward feet movement–with the legs being maximally flexed at the beginning–and the leg extension.
- *leg insweep*, with duration LI: phase between the leg extension and the joining of the legs.
- *leg glide*, with duration LG: phase between the legs joining and the beginning of both the feet moving forward with knee flexion.
- *first part of the leg recovery*, with duration LREC1: phase between the end of the glide and the leg recovery up to a thigh/leg angle of 90 deg.
- *second part of the leg recovery*, with duration LREC2: phase between the end of the first part of the recovery and the complete flexion of the knee until the forward movement of the feet ends up.

Furthermore, four temporal gaps between the stroke phases of each limb, namely, T1–T4, can be identified in Figure 1. They determine the level of arm and leg coordination.

### 2.2. From walking to breast-stroke: self-similarity by transposition

The aforementioned research directions, which have recently provided a theoretical foundation for the appearance of rhythmic motor patterns with hidden time-harmonic structures in front-crawl and butterfly swimming, have started from the walking gait analysis and have proceeded by transposition. Self-similarity increases with the swimming technique, while enhanced self-similarity is linked to top-level swimmers. This study applies the same concise logic.

With this purpose, we start from the walking and recall from Verrelli et al. (2021a) the following facts.

**Figure 2:**
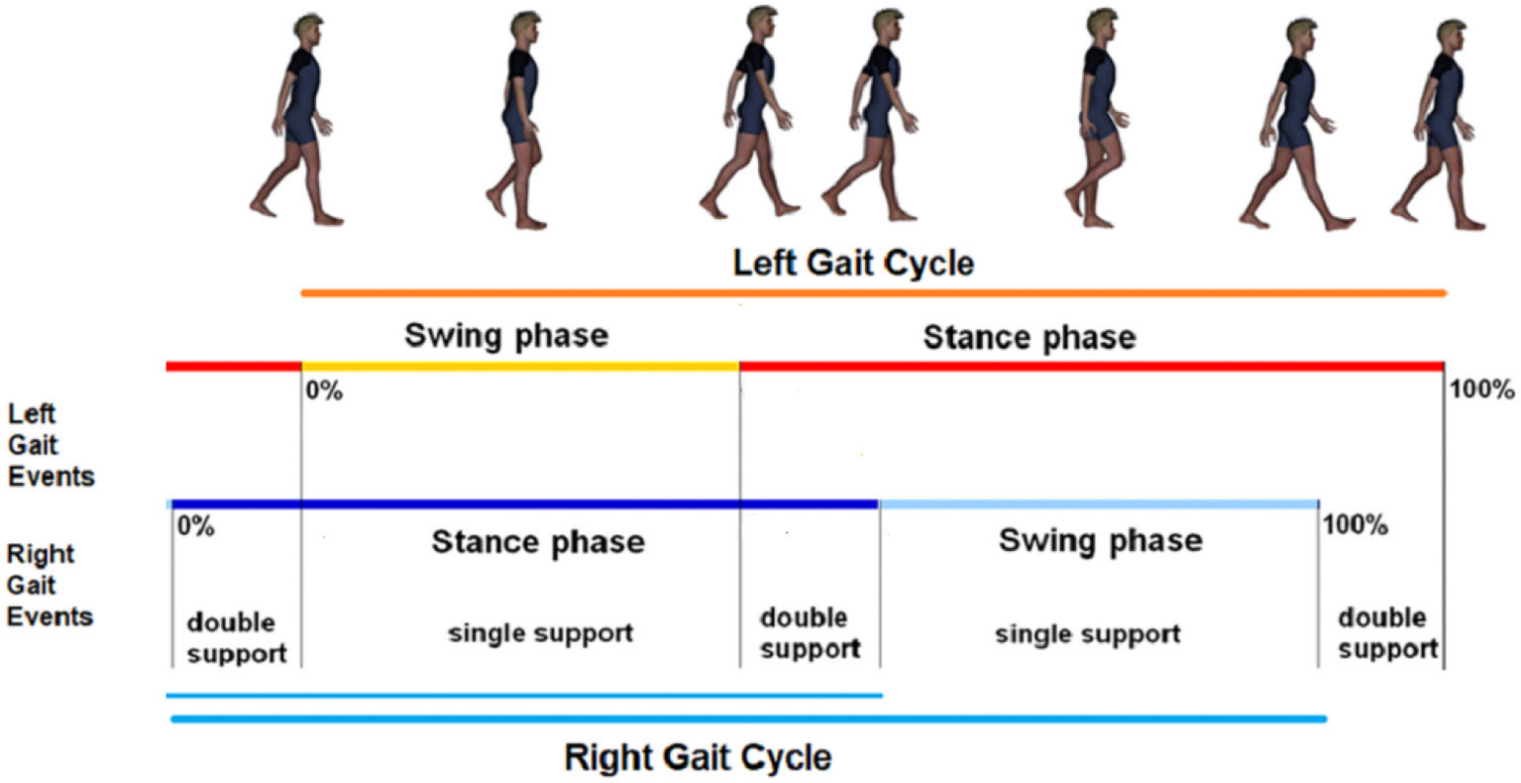
Partitions of the walking gaits.

- Four time intervals—associated with the durations of gait cycle (GC), swing (SW), stance (ST), and double support (DS) phases—temporally describe symmetric and recursive human walking (Dugan and Bat, 2005) (see Figure 2).
- Human walking can be viewed as an expression of generalized finite-length Fibonacci sequences (Horadam, 1961), according to Proposition 2 of Verrelli et al. (2021a):
  *The chain DS* → *SW* → *ST* → *GC represents a (generalized)* (*a, b*)*-generated 4-length Fibonacci sequence of the form: a*, *b*, *c*, *d, with a*, *b*, *c*, *d being non-negative numbers such that c* = *a* + *b and d* = *b* + *c*,
  According to Horadam (1961), the golden ratio ϕ is a fixed point for the consecutive ratios *b*/*a*, *c*/*b* and *d*/*c*.^2^
- The ratio between SW and DS has been experimentally found out in the study mentioned in Iosa et al. (2013) to be approximately equal, in healthy subjects symmetrically and recursively walking at 4 km/h (Cavagna and Margaria, 1966), to the golden ratio $\phi =\left(1+\sqrt{5}\right)/2\approx 1.618$. In other words, since the irrational number ϕ – namely, the positive solution to the equation *x*^2^ = 1 + *x* – comes from the Euclid's problem of cutting in a self-proportional way a given straight segment (Iosa et al., 2017, 2019), ϕ expresses self-similarity in symmetric walking (Iosa et al., 2019). Indeed, in contrast to patients with Parkinson's Disease (Iosa et al., 2016), the foot off event happens at 60–62%(recall that, by definition, 1/ϕ = ϕ − 1) of a physiological gait when the subject is (symmetrically and recursively) walking at a comfortable speed. Therefore, the presence of the golden ratio unveils a fractal nature, in which the larger scale structure resembles the subunit structure and a self-referential loop is generated. As reported in the study mentioned in Igamberdiev (2004), recursive limits lead to canons of perfection.
- The experimental conjecture within the study mentioned in Verrelli et al. (2021a), which extends the ideas underlying a fractal approach to the double support phase within the gait, is inspired by the experimental results reported in the study mentioned in Novacheck (1998) showing that physiological symmetric walking is also characterized by an instant of minimum angular position (with negative sign) of the foot relative to the tibia (with a 90 deg-angle between foot and tibia being plotted at 0 deg)–defining sub-phases of the double support with durations *z*_1_, *z*_2_–occurring at ~7% of gait cycle duration in each double support sub-phase (with 5% as percentage for the complementary interval duration). It is interesting to recognize the structure of a Fibonacci sequence (with fixed point ϕ) there, namely, 5 × 2 = 10 (1/ϕ^5^ ≈ 9.018); 7 × 2 = 14 (1/ϕ^4^ ≈ 14.591); 24 (1/ϕ^3^ ≈ 23.608); 38 (1/ϕ^2^ ≈ 38.198); 62(1/ϕ ≈ 61.804); 100, representing the self-similar representation of the sequence: *a* − (*z*_1_ + *z*_2_), *z*_1_ + *z*_2_, *a*, *b*, *c*, *d*, under the constraint $C_{w}^{\prime }:z_{1}+z_{2}+a=b$.
- A quantitative index, referred to as ϕ*-bonacci gait number*, is defined, which takes its minimum zero value when the enhanced self-similarity occurs.

### 2.3. Self-similarity transposition in breast-stroke

Consider the upper limbs. Then the following derivations rely on a transposition of the roles of the gait/stroke phases from walking to swimming. With respect to this, take REC (equal to REC1 + REC2) in breast-stroke as the transpose of DC in walking (the same analogy can be drawn for the front-crawl and the butterfly strokes of Verrelli et al., 2021b,c). Then, the duration of the complement of the recovery phase in the stroke is to be partitioned into two phases $F_{A}$ and $F_{B}$ with equal duration FA and FB (constraint C below). Such phases are chosen as the $F_{A}=$ *propulsion* phase and as $F_{B}=$ the aggregate *glide* & *elbow push* phase. They are the transpose of the swing phases belonging to the right and left gait in walking, which have the same duration in symmetric walking. Then, the aforementioned Proposition 2 of Verrelli et al. (2021a) is translated into:

Proposition 2.1. *Under the constraint:*


(1) $$\begin{array}{l} C\;:\text{ FA}=\text{FB}, \end{array}$$

*the chain:*


(2) $$\begin{array}{l} \text{REC}\to \text{FA}\to \text{FB}+\text{REC}\to S \end{array}$$

*represents a (generalized)* (*a, b*)*-generated 4-length Fibonacci sequence of the form: a*, *b*, *c*, *d, with a*, *b*, *c*, *d being non-negative numbers such that c* = *a* + *b and d* = *b* + *c. Indeed, when b*/*a* = ϕ*, the stroke is self-similar since b*/*a* = *c*/*b* = *d*/*c* = ϕ*, leading to* FB+REC ≈ 61.804% of S, FA ≈ 38.198% of S, REC ≈ 23.608% of S.

The following proposition (the proof is analogous to the one of Theorem 2 in the study mentioned in Verrelli et al., 2021c) characterizes a stronger self-similarity property, referred to as *enhanced self-similarity*. It is the transpose of the experimental conjecture of the study mentioned in Verrelli et al. (2021a), with min{REC1, REC2} and max{REC1, REC2} being the transpose of *a* − (*z*_1_ + *z*_2_) and *z*_1_ + *z*_2_. It, thus, relies on the transpose of the constraint $C_{w}^{\prime }$, namely,


(3) $$\begin{array}{l} C^{\prime }\;:\text{ REC}+\max\left\{\text{REC}1,\text{REC}2\right\}=\text{FA}. \end{array}$$

Proposition 2.2. *Under the constraints* C *and* $C^{\prime }$*, the chain:*


(4) $$\begin{array}{l} \min\left\{\text{REC}1,\text{REC}2\right\}\to \max\left\{\text{REC}1,\text{REC}2\right\}\to \text{REC}\to \text{FA}\\ \;\to \text{FB}+\text{REC}\to S \end{array}$$

*represents a (generalized)* (*a, b*)*-generated 6-length Fibonacci sequence of the form: a*, *b*, *c*, *d*, *e*, *f, with a*, *b*, *c*, *d*, *e*, *f being non-negative numbers such that c* = *a* + *b*, *d* = *b* + *c*, *e* = *c* + *d*, *f* = *d* + *e. Indeed, when b*/*a* = ϕ*, the stroke possesses an enhanced self-similarity since b*/*a* = *c*/*b* = *d*/*c* = *e*/*d* = *f*/*e* = ϕ*, leading to* FB+REC ≈ 61.804% of S, FA ≈ 38.198% of S, REC ≈ 23.608% of S, max{REC1, REC2} ≈ 14.591% of S, min{REC1, REC2} ≈ 9.0175% of S.

Now, according to our experimental evidence, in the very special case of T3 = max{REC1, REC2}-min{REC1, REC2}, the above sequence, once it is enforced with T3 to the left, becomes a generalized Fibonacci sequence of length 7, with the equality min{REC1, REC2}/T3 = ϕ, making the resulting sequence possess a *strongly enhanced self-similar structure*. In this case, T3 ≈ 5.5735% of S is additionally obtained.

### 2.4. Quantitative measures of self-similarity and enhanced self-similarities in breast-stroke

Three indices $I_{f,4}$, $I_{f,6}$, $I_{f,7}$—named ϕ*-bonacci breast-stroke number, enhanced* ϕ*-bonacci breast-stroke number, and strongly enhanced* ϕ*-bonacci breast-stroke number*—can be naturally introduced in accordance with the previous two Propositions and the last sentence of the previous subsection, in order to quantitatively assess self-similarity, enhanced self-similarity, and strongly enhanced self-similarity of breast-strokes. They are, in order, as follows:


(5) $$\begin{array}{l} I_{f,4}=100[{\left(\left(\text{REC}+\text{FB}\right)/\text{S}-0.61804\right)}^{2}+{\left(\text{FA}/\text{S}-0.38198\right)}^{2}\\ \;{+{\left(\text{REC}/\text{S}-0.23608\right)}^{2}]}^{1/2} \end{array}$$

(6) $$\begin{array}{l} I_{f,6}=100[{\left(\left(\text{REC}+\text{FB}\right)/\text{S}-0.61804\right)}^{2}+{\left(\text{FA}/\text{S}-0.38198\right)}^{2}\\ \;+{\left(\text{REC}/\text{S}-0.23608\right)}^{2}+{\left(\max\left\{\text{REC}1,\text{REC}2\right\}/\text{S}-0.14591\right)}^{2}\\ \;{+{\left(\min\left\{\text{REC}1,\text{REC}2\right\}/\text{S}-0.090175\right)}^{2}]}^{1/2} \end{array}$$

(7) $$\begin{array}{l} I_{f,7}=100[{\left(\left(\text{REC}+\text{FB}\right)/\text{S}-0.61804\right)}^{2}+{\left(\text{FA}/\text{S}-0.38198\right)}^{2}\\ \;+{\left(\text{REC}/\text{S}-0.23608\right)}^{2}+{\left(\max\left\{\text{REC}1,\text{REC}2\right\}/\text{S}-0.14591\right)}^{2}\\ \;{+{\left(\min\left\{\text{REC}1,\text{REC}2\right\}/\text{S}-0.090175\right)}^{2}+{\left(\text{T}3/\text{S}-0.055735\right)}^{2}]}^{1/2}. \end{array}$$

The smaller such indices are, the stronger the corresponding level of self-similarity is. The just *self-similarity* leads to a non-zero value for $I_{f,6}$ that tends to zero when the self-similarity tends to turn into the *enhanced self-similarity*. The same happens for the *enhanced self-similarity* with respect to the *strongly enhanced self-similarity*.

### 2.5. Leg self-similarity in breast-stroke

Consider the leg partition of the breast-stroke and define *x* = LREC1+LREC2 as the duration of the aggregate *first part of the leg recovery* & *second part of the leg recovery*, whereas *y* = LP+LI denotes the duration of the aggregate *leg propulsion* & *leg insweep* phase with *z* = LG being the duration of the *leg glide*. Obviously, *x* + *y* + *z* equals the duration S_l_ of the leg stroke. Define an *equi-partitioned leg stroke* as the one satisfying the constraint:


(8) $$\begin{array}{l} C_{\text{l}}\;:\;x=y. \end{array}$$

For such a stroke—characterizing, in light of our experimental evidence, a swimmer diving over the wave through a sort of rear-traction and not satisfying the arm constraint C—the following Proposition holds true, with the (leg) *recovery* phase still constituting the phase characterizing one of the sequence generators.

Proposition 2.3. *Under the constraint* $C_{\text{l}}$*, the chain:*


(9) $$\begin{array}{l} \min\left\{2\text{x},\text{z}\right\}\to \max\left\{2\text{x},\text{z}\right\}\to 2\text{x}+\text{z} \end{array}$$

*represents a (generalized)* (*a, b*)*-generated 3-length Fibonacci sequence of the form: a*, *b*, *c, with a*, *b*, *c being non-negative numbers such that c* = *a* + *b. Indeed, when b*/*a* = ϕ*, the equi-partitioned leg stroke is self-similar since b*/*a* = *c*/*b* = ϕ*, leading to* max{2*x, z*} ≈ 61.804% of S_l_, min{2*x, z*} ≈ 38.198% of S_l_.

The corresponding index $I_{f,3}$ – named ϕ*-bonacci breast-stroke leg number* – reads:


(10) $$\begin{array}{l} I_{f,3}=100[{\left(\max\left\{x+y,z\right\}/{\text{S}}_{\text{l}}-0.61804\right)}^{2}\\ \;{+{\left(\min\left\{x+y,z\right\}/{\text{S}}_{\text{l}}-0.38198\right)}^{2}]}^{1/2}. \end{array}$$

The smaller such index is, the stronger the corresponding level of leg self-similarity is.

### 2.6. Data collection

The feasibility of the preceding analysis is here illustrated by the dedicated analysis of breast-stroke training sessions for 10 international-level swimmers, namely, IL1 (male, 19 yrs, 188 cm, 85 kg), IL2 (female, 18 yrs, 170 cm, 70 kg), IL3 (male, 23 yrs, 187 cm, 94 kg), IL4 (male, 34 yrs, 188 cm, 82 kg), IL5 (male, 25 yrs, 178 cm, 75 kg), IL6 (male, 33 yrs, 185 cm, 76 kg), IL7 (female, 25 yrs, 170 cm, 55 kg), IL8 (female, 29 yrs, 173 cm, 60 kg), IL9 (female, 27 yrs, 172 cm, 59 kg), and I10 (female, 27 yrs, 162 cm, 57 kg). Such international-level swimmers compete on a regular basis at major international events and hold national/international records. The 2D video analysis is performed by using high frame rate videos (camera: GoPro Hero8, 120 Hz-sample frequency) of stable strokes *via* the BioMovie ERGO system at *http://www.infolabmedia.eu/*.

## 3. Results

The stroke durations for IL1–IL10 are presented in Table 1 (all of the swimmers are required to swim at their own race pace).

**Table 1:** Arm and leg stroke durations (s).

	**Arm**	**Leg**
IL1	0.808	0.866
IL2	0.94	0.98
IL3	1.01	1.04
IL4	0.89	0.94
IL5	0.817	0.851
IL6	1.51	1.627
IL7	1.376	1.394
IL8	1.026	1.034
IL9	1.284	1.393
IL10	1.801	1.944

On the other hand, while swimmers IL1–IL5 exhibit a front-traction, swimmers IL6–IL10 are characterized by a rear-traction. Furthermore, the ranking in terms of physical shape (measured as race performance capabilities) and swimming quality/technique (in terms of aesthetics) views, at the moment of data acquisition, IL3, IL2, and IL10 preceding the remaining ones. The arm phase durations (as percentages with respect to the arm stroke) and the leg phase durations (as percentages with respect to the leg stroke) are presented in Tables 2, 3.

**Table 2:** Arm phase durations (percentages with respect to the arm stroke).

	**T3/S**	**REC1/S**	**REC2/S**	**REC/S**	**FA/S (*%*)**	**(FB + REC)/S (*%*)**
IL1	5.75	11.39	13.37	24.75	37.12	62.87
IL2	4.46	11.46	12.42	23.89	38.11	61.89
IL3	4.16	9.91	13.18	23.09	38.85	61.15
IL4	3.70	11.20	12.09	23.29	37.40	62.60
IL5	3.27	12.24	13.22	25.46	34.76	65.24
IL6	7.74	6.09	7.75	13.84	33.11	66.89
IL7	1.59	7.27	12.71	20.00	31.54	68.46
IL8	0.78	13.84	16.28	30.12	26.80	73.20
IL9	10.70	9.74	15.58	25.31	24.69	75.31
IL10	14.07	7.38	12.05	19.43	26.82	73.18

**Table 3:** Leg phase durations (percentages with respect to the leg stroke).

	***y*/S (*%*)**	***z*/S (*%*)**	***x*/S (*%*)**
IL1	30.72	46.19	23.09
IL2	25.72	51.43	26.57
IL3	29.56	37.62	32.82
IL4	30.92	42.51	26.57
IL5	35.37	40.19	24.44
IL6	23.05	55.38	21.57
IL7	22.17	57.46	20.37
IL8	28.24	43.52	28.24
IL9	27.57	49.68	22.76
IL10	19.34	61.78	18.88

The corresponding self-similarity indices $I_{f,7}$, $I_{f,6}$, $I_{f,4}$, along with the C,$C_{\text{l}}$-constraints evaluation, are presented in Table 4. Since both | T3-max{REC1, REC2}+min{REC1, REC2}|/S, |REC+ max{REC1, REC2} - FA |/S are smaller than 0.04 for IL1-IL5 (not reported here for the sake of brevity), the evaluation of $I_{f,7}$, $I_{f,6}$ will make sense for IL1–IL5.

**Table 4:** Self-similarity indices and C,$C_{\text{l}}$-constraints evaluation (percentages with respect to the corresponding stroke).

	** $I_{f,7}$ **	** $I_{f,6}$ **	** $I_{f,4}$ **	** $I_{f,3}$ **	**(FA − FB)/S (*%*)**	**(*y* − *x*)/S (*%*)**
IL1	0.0328	0.0327	0.0190	0.1130	−0.99	7.63
IL2	0.0347	0.0328	0.0030	0.1630	0.11	−0.85
IL3	0.0243	0.0198	0.0106	0.0082	0.79	−3.26
IL4	0.0399	0.0351	0.0117	0.0610	−1.90	4.35
IL5	0.0668	0.06269	0.0520	0.0282	−5.02	10.93
IL6	0.1439	0.1423	0.1213	0.0909	−19.93	1.48
IL7	0.1114	0.1040	0.1009	0.0614	−16.93	1.80
IL8	0.1989	0.1922	0.19180	0.0753	−16.28	0
IL9	0.1874	0.1811	0.1738	0.1623	−25.31	4.81
IL10	0.1891	0.1690	0.1662	0.0003	−26.93	0.46

According to the previous Tables, comments are in order.

- While IL1–IL5 turn out to satisfy the C constraint, IL6–IL10 satisfy the $C_{\text{l}}$ constraint, thus confirming the front-traction or rear-traction nature of the swimming technique.
- Rather small values for all the self-similarity indices $I_{f,7}$, $I_{f,6}$, and $I_{f,4}$ are obtained for IL1–IL5, with: (i) the IL3's $I_{f,7}$ and $I_{f,6}$ being the smallest ones, owing to phase percentage values close to ≈ 62%, ≈ 38%, ≈ 24%, ≈ 14%, ≈ 9%, and ≈ 5%; (ii) the IL2's $I_{f,4}$ being the smallest ones, owing to phase percentage values close to ≈ 62%, ≈ 38%, and ≈ 24%. While self-similarity is confirmed by the stroke partitions of all the international-level swimmers, the evaluation of the $I_{f,7}$, $I_{f,6}$, and $I_{f,4}$ indices turns out to reproduce the order of physical shape within the two swimmers' set.
- Rather small values for all the self-similarity indices $I_{f,3}$ are obtained for IL6-IL8 and IL10, with the IL10's $I_{f,3}$ being the smallest one, owing to phase percentage values close to ≈ 62 and 38 (notice how, even though IL3 has a small self-similarity index $I_{f,3}$ due to phase percentage values close to ≈ 62 and 38%, the $C_{\text{l}}$ constraint is not fully satisfied for him). On the other hand, IL8, who recently practiced to better perform a rear-traction, exhibits a fully satisfied constraint $C_{\text{l}}$ but not yet high levels of self-similarity.
- The role of the middle/long distance pace in front-crawl and butterfly swimming is here played by the rear-traction.

## 4. Discussion

### 4.1. Discussion of findings

Expanding, by transposition, the connection between human walking and running gaits and front-crawl and butterfly swimming strokes (Marino et al., 2020; Verrelli et al., 2021a,b,c) with the golden ratio (Iosa et al., 2013, 2017), we have found a harmonic characterization of breast-stroke through (simple and enhanced) self-similarity. The presented experimental results, which are exhibited by elite swimmers, confirm how, differently from walking, the swimming technique is relevantly involved: to become expert swimmers, subjects must undergo a considerable amount of practice and instruction (Schiffman et al., 2009; Barbosa et al., 2017; Verrelli et al., 2021b). As a consequence, self-similarity (in its simple and enhanced versions), as defined in this study, might become a reference feature for advanced training programs while not affecting the peculiar stroke duration of the breast-stroke swimmer. Improvements concerning muscle strength act at a different level of analysis. The findings of this study, which even characterize the front-traction or rear-traction nature of the swimming technique, might also introduce comparative (quantitative) information about the functioning of the rhythmic neural patterns during contingent moment-based swimming actions while monitoring improvements over time. Anyway, the present analysis just focuses on the temporal proportions among the stroke phases, with no explicit characterization of the inter-limb coordination (Persyn et al., 1988; Soares et al., 1999) in the case of simple self-similar patterns.

### 4.2. Future directions (back-stroke)

The back-stroke phases can be identified in the study mentioned in Chollet et al. (2008) (Figure 1 therein) as follows:

- *entry and catch* of the hand in the water, with duration EC: phase between the entry of the hand into the water and the beginning of its backward movement that is followed by a diagonal hand sweep.
- *pull*, with duration PL: phase between the beginning of the hand backward movement (followed by a diagonal hand sweep) and its arrival in a plane vertical to the shoulder (first part of propulsion).
- *push*, with duration PS: phase from the position of the hand below the shoulder to the end of the hand backward movement.
- *hand lag time*, with duration HLT: phase during which the hand stops at the thigh after the push phase and before the clearing.
- *clearing*, with duration CL: phase between the beginning of the hand release and the beginning of its exit from the water.
- *recovery*, with duration REC: phase corresponding to the point of water release to water re-entry of the arm, i.e., the above-water phase.

The sum of the above durations provides the duration S of the stroke. Notice how in Chollet et al. (2008) propulsion has not been considered as hand-force production. Instead, it is conceived as a voluntary act to propel the body forward. The duration of the propulsive phase is, thus, the sum of the *pull* and *push* phase durations, while the duration of the non-propulsive phase is the sum of the *entry and catch, hand lag time, clearing*, and *recovery* phase durations.

Since back-stroke is actually similar to front-crawl swimming, we start from the front-crawl stroke analysis of the study mentioned in Verrelli et al. (2021b) and again proceed by transposition. Therefore, as in the study mentioned in Verrelli et al. (2021b), first, we divide the back-stroke into the *out-of-water* phase (coinciding with the *recovery* phase) and *in-water* phase, that is the complement of the *out-of-water* phase within the stroke. The duration of the *recovery* phase is the generator of the sequence as in the breast-stroke. Then, we take the aggregate *pull* & *push* phase within the *in-water* phase, which constitutes the propulsive phase (underlying the aforementioned meaning). This aggregate phase is the mirrored counterpart of the strong propulsive phase of Remark 1 of Verrelli et al. (2021b) and constitutes our aggregate phase $F_{A}$ (in analogy with the breast-stroke) with duration FA. Its complement within the *in-water* phase, that is the aggregate *entry and catch* & *hand lag time* & *clearing* phase, is our $F_{B}$ (in analogy with the breast-stroke) with duration FB. Both of them are assumed to satisfy the C constraint as in the front-crawl and the breast-stroke. The following proposition, which is the perfectly equivalent version of Proposition 2.1 for back-stroke, thus naturally holds.

Proposition 4.1. *Under the constraint:*


(11) $$\begin{array}{l} C_{\text{b}}\;:\text{ FA}=\text{FB}, \end{array}$$

*the chain:*


(12) $$\begin{array}{l} \text{REC}\to \text{FA}\to \text{FB}+\text{REC}\to S \end{array}$$

*represents a (generalized)* (*a, b*)*-generated 4-length Fibonacci sequence of the form: a*, *b*, *c*, *d, with a*, *b*, *c*, *d being non-negative numbers such that c* = *a* + *b and d* = *b* + *c. Indeed, when b*/*a* = ϕ*, the stroke is self-similar since b*/*a* = *c*/*b* = *d*/*c* = ϕ*, leading to* FB+REC ≈ 61.804% of S, FA ≈ 38.198% of S, REC ≈ 23.608% of S.

### 4.3. Conclusion

This study has extended—on the basis of experimental results involving international-level breast-stroke swimmers—the recent findings on self-similar pattern-based quantitative indices for front-crawl/butterfly strokes to the remaining two swimming strokes, namely, back-stroke and the breast-stroke. The relevance of such an extension can be hardly underestimated. Harmonic structures might play the roles of reference points in training programs for all the swimming stroke types (with, anyway, no limitations of the individual stroke duration) while providing comparative (quantitative) information about both the physical recovery level (after an intense workout) and the functioning of rhythmic neural patterns (in any stroke type) during a specific swimming performance. Finally, the results of this study seek to open new research directions that aim at associating computational number theory with neural system modulating principles in swimming.

## Data availability statement

The original contributions presented in the study are included in the article, further inquiries can be directed to the corresponding author.

## Ethics statement

This study was reviewed and approved by The Internal Research Board (University of Rome Tor Vergata). Written informed consent was obtained from the individuals for the publication of any potentially identifiable images or data included in this article.

## Author contributions

CV and CR: conceptualization. CV, CR, IF, GA, and VB: methodology. CR and IF: software and resources. CV, CR, and IF: validation, investigation, and formal analysis. CV and NC: writing—original draft. CV, CR, and VM: writing—reviewing and editing. All authors contributed to the article and approved the submitted version.

## Footnotes

^1^Notice how the different partition of Takagi et al. (2004), involving a slightly different definition of the *recovery phase*, was at the root of the considerations of Section 7 of Verrelli et al. (2021c), which are, in turn, perfectly compatible with the analysis of this paper.

^2^In fact, *b*/*a* = ϕ implies: *c*/*b* = (*a* + *b*)/*b* = 1/ϕ + 1 = ϕ and *d*/*c* = (*b* + *c*)/*c* = 1/ϕ + 1 = ϕ. In such a case, the sequence is obeys: *y*(*k* + 1) = ϕ*y*(*k*), *k* = 0, 1, 2, where *y*(0) = *a*, *y*(1) = *b*, *y*(2) = *c*, *y*(3) = *d*. One single value thus *determines* the whole sequence (see the Shannon entropy- based interpretation of Verrelli et al., 2021a).
